# Indoxyl Sulfate-Induced Activation of (Pro)renin Receptor Promotes Cell Proliferation and Tissue Factor Expression in Vascular Smooth Muscle Cells

**DOI:** 10.1371/journal.pone.0109268

**Published:** 2014-10-24

**Authors:** Maimaiti Yisireyili, Shinichi Saito, Shaniya Abudureyimu, Yelixiati Adelibieke, Hwee-Yeong Ng, Fuyuhiko Nishijima, Kyosuke Takeshita, Toyoaki Murohara, Toshimitsu Niwa

**Affiliations:** 1 Department of Advanced Medicine for Uremia, Nagoya University Graduate School of Medicine, Nagoya, Japan; 2 Biomedical Research Laboratories, Kureha Co., Tokyo, Japan; 3 Department of Cardiology, Nagoya University Graduate School of Medicine, Nagoya, Japan; 4 Division of Nephrology, Department of Internal Medicine, Kaohsiung Chang Gung Memorial Hospital and Chang Gung University College of Medicine, Kaohsiung, Taiwan; 5 Faculty of Health and Nutrition, Shubun University, Aichi, Japan; Medical University Innsbruck, Austria

## Abstract

Chronic kidney disease (CKD) is associated with an increased risk of cardiovascular disease (CVD). (Pro)renin receptor (PRR) is activated in the kidney of CKD. The present study aimed to determine the role of indoxyl sulfate (IS), a uremic toxin, in PRR activation in rat aorta and human aortic smooth muscle cells (HASMCs). We examined the expression of PRR and renin/prorenin in rat aorta using immunohistochemistry. Both CKD rats and IS-administrated rats showed elevated expression of PRR and renin/prorenin in aorta compared with normal rats. IS upregulated the expression of PRR and prorenin in HASMCs. N-acetylcysteine, an antioxidant, and diphenyleneiodonium, an inhibitor of nicotinamide adenine dinucleotide phosphate oxidase, suppressed IS-induced expression of PRR and prorenin in HASMCs. Knock down of organic anion transporter 3 (OAT3), aryl hydrocarbon receptor (AhR) and nuclear factor-κB p65 (NF-κB p65) with small interfering RNAs inhibited IS-induced expression of PRR and prorenin in HASMCs. Knock down of PRR inhibited cell proliferation and tissue factor expression induced by not only prorenin but also IS in HASMCs.

**Conclusion:**

IS stimulates aortic expression of PRR and renin/prorenin through OAT3-mediated uptake, production of reactive oxygen species, and activation of AhR and NF-κB p65 in vascular smooth muscle cells. IS-induced activation of PRR promotes cell proliferation and tissue factor expression in vascular smooth muscle cells.

## Introduction

Patients with chronic kidney disease (CKD) are at high risk for cardiovascular disease (CVD). CKD leads to accelerated atherosclerosis and consequently to a marked increase in cardiovascular morbidity and mortality [1]. Accumulation of indoxyl sulfate (IS), a protein-bound uremic toxin, is involved in the progression of not only CKD, but also CVD [2]–[4]. IS is a metabolite of tryptophan derived from dietary protein, and is synthesized in the liver from indole that is produced by intestinal flora including *Escherichia coli*. IS is normally excreted into urine. As renal function deteriorates, IS accumulates in serum due to its reduced renal clearance [2], [5]. Because of its protein binding ability, removal by hemodialysis is not as efficient as that of non-protein bound uremic toxin. IS shows nephrotoxicity after its uptake by renal proximal tubular cells through the basolateral membrane via organic anion transporter 1 (OAT1) and OAT3 [6].

IS induces reactive oxygen species (ROS) and activates nuclear factor-κB (NF-κB) [7], p53 [8], and signal transducer and activator of transcription 3 (Stat3) [9]. Then, IS stimulates renal expression of fibrotic genes such as transforming growth factor-β1 (TGF-β1) [7], [10], and α-smooth muscle actin (SMA) [7], [8], and inflammatory genes such as monocyte chemotactic protein-1 (MCP-1) and intercellular adhesion molecule-1 (ICAM-1) in proximal tubular cells [11], [12]. These pathophysiological changes facilitate kidney dysfunction such as interstitial fibrosis and inflammation, accelerating the progression of CKD [2], [3].

Besides its negative effect on kidney, serum level of IS was associated with CVD and mortality in CKD patients [13]. IS promoted aortic calcification and senescence in hypertensive rats [14], and induced dysfunction of vascular endothelial cells [15], [16], vascular smooth muscle cells [17], [18], and cardiomyocytes [19], [20]. Thus, IS is a nephrovascular uremic toxin [3].

Renin angiotensin system (RAS) plays an important role in CKD. (Pro)renin receptor (PRR), which binds to both renin and prorenin, is a newly discovered component of RAS, and is highly expressed not only in the kidney, but also in cardiovascular system [21]–[23].

Prorenin bound to PRR becomes enzymatically active, and can catalyze angiotensinogen into angiotensin (Ang) I. Further, PRR bound to prorenin or renin induces intracellular signaling and activation of mitogen-activated protein kinase (MAPK) ERK1/2, leading to activation of TGF-β1, independent of Ang II production [21], [24]. PRR is expressed in the subendothelium of coronary arteries and, more precisely, in vascular smooth muscle cells [22]. Upregulation of PRR is involved in renal fibrosis [21], [25], and vascular smooth muscle cell proliferation [22], [26], [27]. Further, recent studies revealed different roles of PRR, linked to the vacuolar H^+^-ATPase (V-ATPase) activity, Wnt signaling, and autophagy [28]–[32]. Thus, PRR activation plays an important role in the pathophysiology of not only CKD but also CVD.

IS induces vascular smooth muscle cell proliferation through ROS and activation of p44/42 MAPK pathway [33], [34]. IS induces tissue factor expression and activity in vascular smooth muscle cells [35]. Tissue factor is a crucial mediator of injury-related thrombosis and vascular smooth muscle cell proliferation, and has been implicated for stent thrombosis observed in patients with advanced CKD [35]. However, the role of IS in PRR expression in vascular smooth muscle cells has not yet been studied.

The present study aimed to clarify whether IS induces PRR expression in rat aortic tissues and human aortic smooth muscle cells (HASMCs), and whether PRR mediates IS-induced cell proliferation and tissue factor expression in HASMCs.

## Methods

### Reagents

Reagents and antibodies were obtained from the following companies: HASMCs were purchased from Cascade Biologics (Portland, OR, USA). Dulbecco's modified Eagle's medium (D-MEM), fetal bovine serum (FBS), penicillin–streptomycin and trypsin-EDTA solutions were purchased from Gibco (Invitrogen, Grand Island, NY, USA). IS was from Sigma Chemical (St. Louis, MO, USA). N-acetylcyteine (NAC), an antioxidant, and diphenyleneiodonium chloride (DPI), an inhibitor of nicotinamide adenine dinucleotide phosphate (NADPH) oxidase, were obtained from Calbiochem (La Jolla, CA, USA). Human prorenin was obtained from Molecular Innovations (Novi, MI, USA). Anti-PRR and anti-prorenin antibodies were from Abcam (Cambridge, UK). Anti-renin/prorenin antibody used for immunohistochemistry, which cross-reacts with renin and prorenin [36], was kindly provided by Tadashi Inagami (Department of Biochemistry, Vanderbilt University School of Medicine, Nashville, TN, USA). Anti-NF-κB p65, anti-aryl hydrocarbon receptor (AhR), and anti-tissue factor antibodies were from Santa Cruz Biotechnology (Santa Cruz, CA, USA). Anti-α-tubulin was from Calbiochem (La Jolla, CA, USA). Anti-rabbit IgG horseradish peroxidase (HRP)-linked antibody and anti-mouse IgG HRP-linked antibody were from Cell Signaling Technology (Beverly, MA, USA). CellTiter 96 Aqueous One Solution Cell Proliferation Assay was from Promega (Madison, WI, USA), and Lipofectamine RNA iMAX reagent was from Invitrogen (Life Technologies, Carsbad, CA, USA).

### Animal Study 1

Experimental rats were prepared as reported previously [37]. Seven-week old male Sprague-Dawley rats (Clea, Tokyo, Japan) were used to produce CKD rats by 5/6- nephrectomy. Eleven weeks after subtotal nephrectomy, the rats were randomized into two groups, control CKD rats (n = 8), and AST-120-treated CKD rats (n = 8). AST-120 was orally administered to the rats at a dose of 4 g/kg/day with powder chow (CE-2, Clea, Tokyo, Japan) for 10 weeks, whereas powder chow alone was administered to control CKD rats. Normal rats (n = 9) were used to compare the data with CKD rats. After administration of AST-120 for 16 weeks, the rats were anesthetized, and arcuate aortas were excised for immunohistochemical study.

### Animal Study 2

Experimental rats were prepared as reported previously [38]. Briefly, the animal groups consisted of: (1) Dahl normotensive rats (DN, n = 8), (2) Dahl normotensive IS-administered rats (DN+IS, n = 8), (3) Dahl hypertensive rats (DH, n = 8), and (4) Dahl hypertensive IS-administered rats (DH+IS, n = 8). IS (200 mg/kg/day in drinking water) was administered to the rats. At 48 weeks of age (32nd week of the study), their arcuate aortas were excised for immunohistochemical analysis. Serum IS levels were measured by high-performance liquid chromatography as reported previously [5]. Blood pressure was measured using the tails of the rats with a pneumatic cuff and a sphygmomanometer for small animals (UR-5000, Ueda Avancer Co., Tokyo, Japan).

The Animal Care Committee of Kureha Biomedical Research Laboratories approved these animal studies, which proceeded according to the Guiding Principles for the Care and Use of Laboratory Animals of the Japanese Pharmacological Society.

### Immunohistochemistry

Immunohistochemistry was performed according to the streptavidin-biotinylated peroxidase complex (SABC) method. Aortic sections were deparaffinized with xylene, and dehydrated with ethanol. Endogenous peroxidase activity was inhibited with 0.3% H_2_O_2_ in methanol at room temperature for 10 min, followed by a rinse with phosphate buffered saline (PBS). All sections were incubated with 10% normal serum at room temperature for 30 min. Heat-mediated antigen retrieval method was performed twice by microwave treatment with 0.01 mol/L citrate buffer (pH 6.0) for 5 min. Then, the sections were treated at 4°C overnight with a primary antibody, anti-PRR antibody (1∶100) or anti-renin/prorenin (1∶50) antibody which cross reacts with renin and prorenin. Then, the sections were incubated with a secondary antibody at room temperature for 30 min followed by a rinse with PBS, and then treated with peroxidase-conjugated streptavidin (Nichirei Co) at 37°C for 30 min. Finally, localization of PRR and renin/prorenin was visualized using 3,3-diaminobenzidine tetrahydrochloride (DAB tablet; Merck KGaA, Darmstadt, Germany) at a concentration of 30 mg/mL, containing 0.03% H_2_O_2_. Then, the sections were counterstained with methylene green, and mounted in mounting media (Mount-quick, Daydo Sangyo Co., Saitama, Japan). All sections were photographed under light microscopy (×400) with digital camera (DN100, E-600, Nikon; Tokyo, Japan). Immunostaining-positive areas were determined using Adobe Photoshop, and quantified in 10 random fields per section using NIH Image 1.62.

## Cell Culture

HASMCs were maintained in D-MEM containing 10% FBS supplemented with 100 U/mL penicillin, 100 µg/mL streptomycin at standard cell culture condition (37°C under 5% CO_2_ humidified atmosphere). The medium was replaced every three days until confluence. Only cells between passages 2 to 8 were used for experiments.

### Measurement of Cell Proliferation

Proliferation of HASMCs was measured using Cell Titer 96 Aqueous One Solution Cell Proliferation Assay [16]. Cells were seeded at a density of 5×10^3^ cells/well on 24 well culture plate in D-MEM containing 10% FBS for 48 h. Serum-starved HASMCs (5×10^3^ cells/well) in a 24-well plate were stimulated with or without IS (250 µmol/L) or prorenin (20 nmol/L) for 24 h. For gene knockdown experiment, HASMCs were transfected with siRNA for PRR (20 nmol/L) for 48 h, before IS or prorenin stimulation. Thereafter, cell proliferation reagent MTS (50 µL) was added to each well, and cells were incubated for 4 h. The absorbance was measured at 492 nm using a microplate reader (DS PharmaBiomedical Co., Ltd, Osaka, Japan).

### Quantitative Real-Time Polymerase Chain Reaction (RT-PCR)

Total RNA was extracted from HASMCs lysates using TRIzol Reagent (Life Technologies, Carlsbad, CA) and subjected to reverse transcription. The cDNA was subjected to a quantitative RT-PCR analysis with the use of a Bio-Rad CFX96 RT-PCR Detection System and Power SYBR Green PCR Master Mix (Applied Biosystems, Foster City, CA). Serial dilutions of a control sample of cDNA were used as the standard curve for each reaction. All experiments were performed in triplicate. Changes in gene expression were normalized the values to the levels of glyceraldehyde 3-phosphate dehydrogenase (GAPDH). Primers (Sigma-Aldrich) used were: PRR, 5′-AAT TGG CCT ATA CCA GGA GAG C-3′ (forward) and 5′-GAA ACA GGT TAC CCA CTG CGA-3′ (reverse); Prorenin, 5′-CCA CCT CCG TGA TCC T-3′ (forward) and 5′-GCG GAT AGT ACT GGG TGT CCA T-3′ (reverse); GAPDH, 5′-ATG GGG AAG GTG AAG GTC G-3′ (forward) and 5′-GGG GTC ATT GAT GGC AAC AAT A-3′ (reverse).

### Transfection of siRNA

Small interfering RNAs (siRNAs) specific to OAT3 (OAT3 siRNA) and AhR (AhR siRNA) were purchased from Santa Cruz Biotechnology (Santa Cruz, CA, USA). siRNAs specific to PRR (PRR siRNA) and NF-κB p65 (NF-κB p65 siRNA) were obtained from Nippon EGT (Tokyo, Japan). Lipofectamine RNA iMAX (Invitrogen, Life Technologies, Carsbad, CA, USA) was used to transfect siRNA into HASMCs cells according to a manufacturer's protocol. HASMCs were incubated with or without OAT3 siRNA (10 nmol/L), AhR siRNA (30 nmol/L), NF-κB p65 siRNA (10 nmol/L) or PRR siRNA (20 nmol/L) for 48 h. Protein expressions of OAT3, AhR, NF-κB p65, and PRR were analyzed by western blotting.

### Western Blot Analysis

Serum-starved HASMCs were incubated with 250 µmol/L of IS for the indicated time periods. Cells were pretreated with 2.5 mmol/L NAC and 10 µmol/L DPI for 30 min, before IS stimulation for 24 h. For gene knock down experiment, HASMCs were transfected with siRNAs (siOAT3, siPRR, sip65 and siAhR) for 48 h, before IS stimulation. Cells were lysed in lysis buffer (65 mmol/L Tris-HCl (PH 6.8), 3.3% sodium dodecyl sulfate (SDS), 10% glycerol, 2.2% bromophenol blue) and were fractionated by SDS-polyacrylamide gel electrophoresis (PAGE) on polyacrylamide gels. Then, proteins were transferred to polyvinylidene difluoride (PVDF) membranes (Immobilon-P, Millipore Bedford, MA, USA). The membranes were blocked with 5% bovine serum albumin (BSA) in Tris-buffered saline tween-20 (TBS-T) at room temperature for 1 h. After washing with TBS-T, the membranes were treated with rabbit polyclonal anti-PRR antibody (1∶1000), rabbit monoclonal anti-prorenin antibody (1∶1000), rabbit polyclonal anti-OAT3 antibody (1∶500), rabbit polyclonal anti-NF-κB p65 antibody (1∶1000), rabbit polyclonal anti-AhR antibody (1∶1000) and goat polyclonal anti-tissue factor antibody (1∶1000), respectively. Then, the membranes were further incubated with HRP-linked secondary antibody (1∶5000) at room temperature for 1 h. After washing with TBS-T three times, the protein expressions were visualized using the enhanced Chemi-Lumi one system (Nacalai Tesque, Kyoto, Japan). The intensity of protein bands normalized to the amount of α-tubulin (an internal control, 1∶1000) is expressed as ratios (fold increase) of the control value.

### Statistical Analysis

Results are expressed as mean±SE. The quantitative data among different groups were analyzed by Fisher's protected least significant difference (PLSD) test of one-way analysis of variance (ANOVA). Results were considered statistically significant when *P* value was <0.05.

## Results

### AST-120 Supresses Aortic Expression of PRR in CKD Rats

An oral absorbent (AST-120, Kremezin, Kureha Co., Tokyo, Japan) reduces serum levels of IS in CKD rats and patients [39]. To examine the effects of AST-120 on PRR expression in aorta, AST-120 was orally administered to CKD rats. Laboratory parameters of the animal study 1 were described previously [37]. Briefly, serum levels of IS were 0.008±0.007 mg/dL in normal rats, 0.52±0.16 mg/dL in CKD rats, and 0.12±0.02 mg/dL in AST-120-treated CKD rats [37].

**Figure 1:**
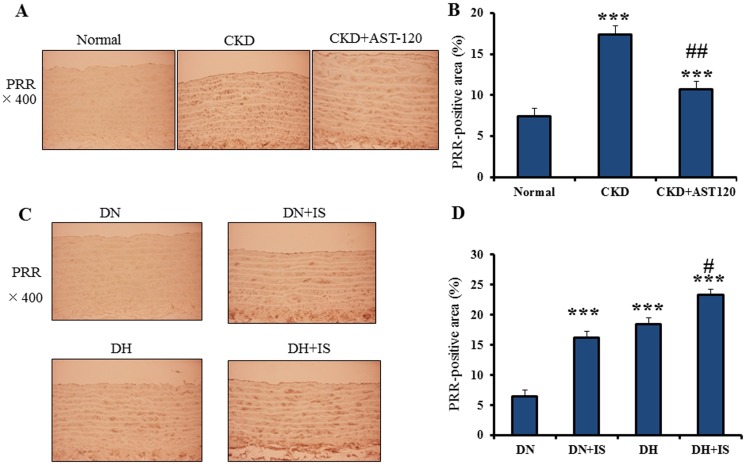
Immunohistochemistry of PRR in rat aorta. **A**. Immonohistochemical localization of PRR in the aortas of normal, CKD and AST-120-treated CKD rats. **B**. Quantitative data of PRR in the aortas of normal (n = 9), CKD (n = 8) and AST-120-treated CKD rats (n = 8) (mean±SE). ***p<0.001 vs normal, ##p<0.001 vs CKD. **C**. Immonohistochemical localization of PRR in the aortas of DN, DN+IS, DH and DH+IS rats. **D**. Quantitative data of PRR-positive area in the aorta of DN, DN+IS, DH and DH+IS rats (mean±SE, n = 8). ***p<0.001vs DN, #p<0.05 vs DH.

CKD rats showed significantly increased expression of PRR in the arcuate aorta compared with normal rats (Figure 1A, B). On the other hand, AST-120-treated CKD rats revealed significantly reduced expression of PRR in the arcuate aorta compared with CKD rats (Figure 1A, B).

### IS Enhances Aortic Expression of PRR in Normotensive and Hypertensive Rats

To determine the effect of IS on PRR expression in aorta, IS was orally administered to normotensive and hypertensive rats. Laboratory parameters of animal study 2 were reported previously [38]. Briefly, serum levels of IS at the 32nd weeks of the study were; 0.10±0.01 mg/dL in DN rats, 0.94±0.13 mg/dL in DN+IS rats, 0.06±0.01 mg/dL in DH rats, and 1.89±0.26 mg/dL in DH+IS rats [38]. Systolic blood pressure levels at the 32nd weeks of the study were; 143±3 mmHg in DN rats, 141±3 mmHg in DN+IS rats, 158±5 mmHg in DH rats, and 158±9 mmHg in DH+IS rats [38].

DN+IS, DH and DH+IS rats showed significantly increased expression levels of PRR in arcuate aorta compared with DN rats (Figure 1C,D). Furthermore, DH+IS rats showed significantly elevated expression level of PRR in arcuate aorta compared with DH rats (Figure 1C,D). Taken together, IS as well as hypertension increased PRR expression in rat aorta.

### Aortic Expression of Renin/prorenin is Increased in CKD Rats and IS-treated Rats

**Figure 2:**
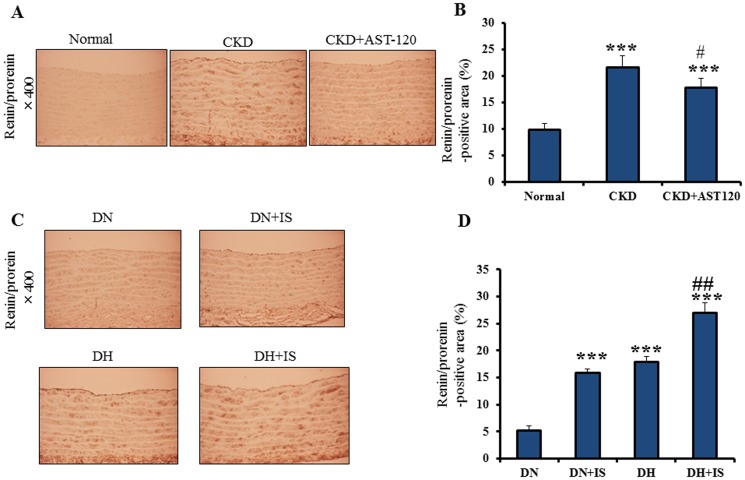
Immunohistochemistry of renin/prorenin in rat aorta. **A**. Immonohistochemical localization of renin/prorenin in the aortas of normal, CKD and AST-120-treated CKD rats. **B**. Quantitative data of renin/prorenin-positive area in the aortas of normal (n = 9), CKD (n = 8) and AST-120-treated CKD rats (n = 8) (Mean±SE). ***p<0.001 vs normal, #p<0.05 vs CKD. **C**. Immonohistochemical localization of renin/prorenin in the aortas of DN, DN+IS, DH and DH+IS rats. **D**. Quantitative data of renin/prorenin-positive area in the aortas of DN, DN+IS, DH and DH+IS rats (mean±SE, n = 8). ***p<0.001vs DN, ##p<0.01 vs DH.

Immunohistochemical analysis was conducted to examine whether IS upregulates renin/prorenin expression in the aorta of CKD rats and IS-treated rats. Anti-renin/prorenin antibody, which cross reacts with renin and prorenin, was used as a primary antibody [36]. Aortic expression of renin/prorenin was significantly increased in CKD rats compared with normal rats. However, AST-120-treated CKD rats reduced the expression of renin/prorenin compared with CKD rats (Figure 2A,B).

Aortic expression of renin/prorenin was significantly increased in DN+IS and DH+IS rats compared with DN and DH rat, respectively (Figure 2C,D). Taken together, CKD rats and IS-administered rats showed increased expression of renin/prorenin in the aorta.

### IS Induces PRR Expression in Vascular Smooth Muscle Cells

**Figure 3:**
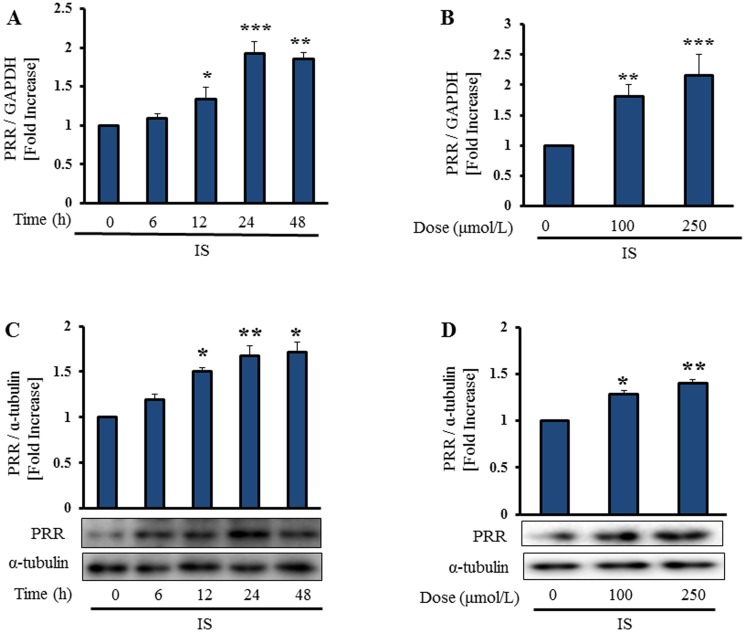
IS induces PRR expression in vascular smooth muscle cells. Serum-starved HASMCs were treated with IS (250 µmol/L). Incubation with IS increased PRR mRNA and protein expression in HASMCs time- (**A**, **C**) and dose- (**B**, **D**) dependently. Mean±SE (n = 3). *p<0.05, **p<0.01, ***p<0.001 vs control.

We confirmed the effect of IS on expression of PRR by incubating HASMCs with IS at indicated time periods and concentrations. IS stimulated expression of PRR mRNA and protein in a time- and dose-dependent manner in HASMCs (Figure 3A–D). The molecular weight of PRR protein was 39 kDa. PRR expression was examined at 24 h after incubating with IS at different concentrations. IS at a concentration of 250 µmol/L was used for the further *in-vitro* study, because it is comparable to the mean serum level of IS in hemodialysis patients [2].

### ROS, OAT3, AhR and NF-κB p65 are Involved in IS-Induced PRR Expression in Vascular Smooth Muscle Cells

**Figure 4:**
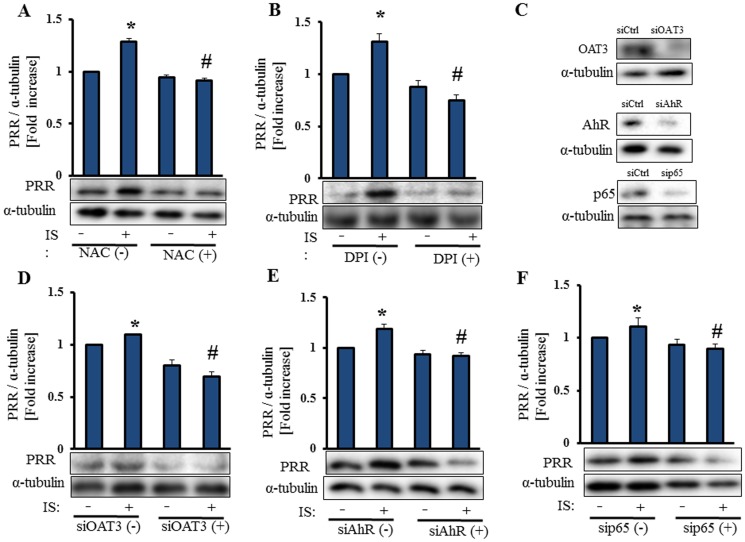
ROS, OAT3, AhR and NF-κB p65 are involved in IS-induced PRR expression in vascular smooth muscle cells. Serum-starved HASMCs were pretreated with NAC (2.5 mmol/L) and DPI (10 µmol/L) for 30 min before incubation with IS (250 µmol/L) for 24 h (A,B). HASMCs were transfected with or without OAT3 siRNA (10 nmol/L), AhR siRNA (30 nmol/L) or p65 siRNA (10 nmol/L), and serum starved for 24 h, followed by incubation with IS (250 µmol/L) for 24 h. Cell lysates were immunoblotted using anti-OAT3, anti-AhR, anti-p65 and anti-PRR antibodies (**C–F**). Mean±SE (n = 3). *p<0.05 vs control, #p<0.05 vs IS-treated group. Ctrl: control.

Serum-starved HASMCs were pre-incubated with 2.5 mmol/L NAC, an antioxidant, or 10 µmol/L of DPI, an inhibitor of NADPH oxidase, and then stimulated with 250 µmol/L IS for 24 h (Figure 4A,B). IS induces ROS production and expression of NADPH oxidase 4 (NOX-4) in HASMCs [16], [17]. Both NAC and DPI suppressed IS-induced protein expression of PRR. Therefore, IS upregulated PRR expression in HASMCs through ROS. IS is transported into HASMCs by OAT3 [6]. OAT3 siRNA suppressed IS-induced expression of PRR in HASMCs (Figure 4D), Thus, IS is taken up by OAT3, and induces PRR expression in HASMCs.

IS was identified as an AhR agonist in human hepatocytes [40], endothelial cells [41], [42], and vascular smooth muscle cells [43]. Activation of AhR mediates IS-induced expression of MCP-1 and tissue factor in endothelial cells [41]. IS activates NF-κB pathway in proximal tubular cells [7] and endothelial cells [14]. We hypothesized that IS-induced expression of PRR and prorenin is mediated by activation of AhR and NF-κB p65. Both AhR siRNA and NF-κB p65 siRNA suppressed IS-induced expression of PRR (Figure 4E,F). Thus, IS induced expression of PRR through activation of AhR and NF-κB p65 in HASMCs.

### IS Induces Prorenin Expression in Vascular Smooth Muscle Cells

**Figure 5:**
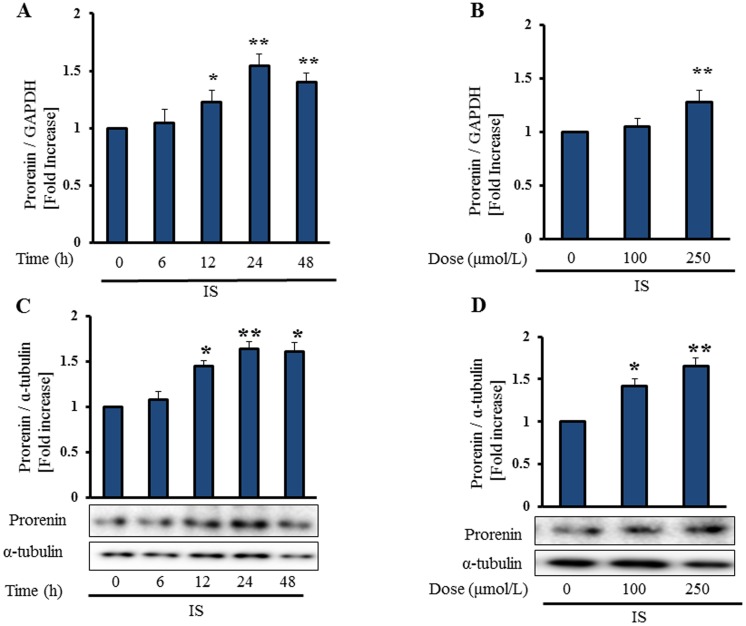
IS induces prorenin expression in vascular smooth muscle cells. Serum-starved HASMCs were treated with IS (250 µmol/L). Incubation with IS increased prorenin mRNA and protein expression in HASMCs time- (**A**, **C**) and dose- (**B**, **D**) dependently. Mean±SE (n = 3). *p<0.05, **p<0.01 vs control.

Prorenin which is bound to PRR, becomes enzymatically active, and then catalyzes angiotensinogen into Ang I. Further, prorenin-bound PRR induces intracellular signaling [21]. However, if prorenin does not exist, PRR could not be activated. Therefore, we examined whether IS induces prorenin expression in HASMCs. IS promoted expression of prorenin mRNA and protein in HASMCs time- and dose-dependently (Figure 5A–D). The molecular weight of prorenin was 47 kDa.

### ROS, OAT3, AhR, and NF-κB p65 are Involved in IS-Induced Prorenin Expression in Vascular Smooth Muscle Cells

**Figure 6:**
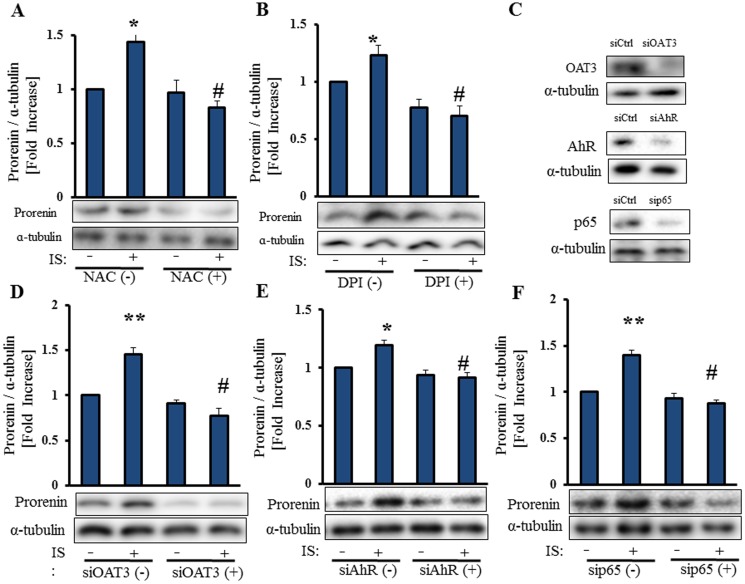
ROS, OAT3, AhR, and NF-κB p65 are involved in IS-induced prorenin expression in vascular smooth muscle cells. Serum starved HASMCs were pretreated with NAC (2.5 mmol/L) and DPI (10 µmol/L) for 30 min before incubation with IS (250 µmol/L) for 24 h (**A, B**). HASMCs were transfected with or without OAT3 siRNA (10 nmol/L), AhR siRNA (30 nmol/L) or p65 siRNA (10 nmol/L), and serum starved for 24 h, followed by incubation with IS (250 µmol/L) for 24 h. Cell lysates were immunoblotted using anti-OAT3, anti-AhR, anti-p65 and anti-prorenin antibodies (**C–F**). Mean±SE (n = 3). *p<0.05, **p<0.01 vs untreated group, #p<0.01 vs IS-treated group. Ctrl: control.

Both NAC and DPI suppressed stimulatory effects of IS on prorenin expression in HASMCs (Figure 6A, B). OAT3 siRNA, AhR siRNA and NF-κB p65 siRNA inhibited stimulatory effects of IS on prorenin expression. (Figure 6D, E, F). Therefore, IS induced prorenin expression in HASMCs through ROS, OAT3, AhR and NF-κB p65.

### IS-Induced PRR Activation is Involved in Vascular Smooth Muscle Cell Proliferation

**Figure 7:**
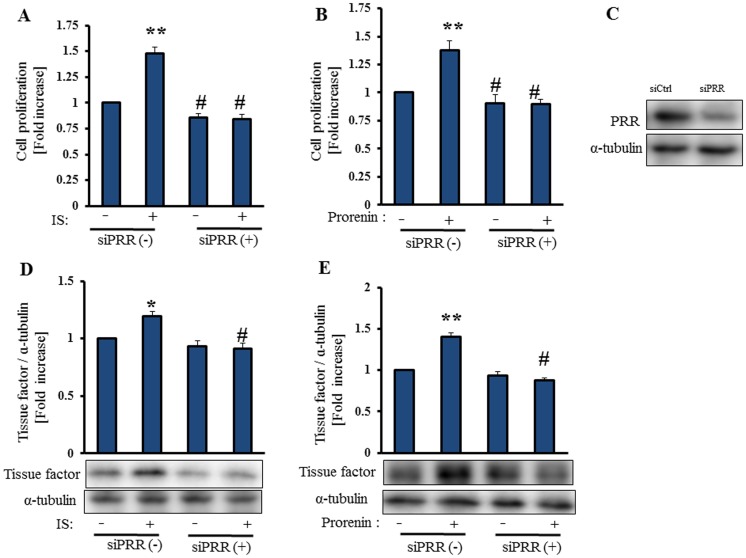
IS-induced PRR activation is involved in cell proliferation and tissue factor expression in vascular smooth muscle cells. Serum-starved HASMCs (5×10^3^ cells/well) in a 24-well plate were stimulated with or without IS (250 µmol/L) or prorenin (20 nmol/L) for 24 h (**A, B**). HASMCs were transfected with siPRR (20 nmol/L) for 48 h, before stimulation with IS or prorenin for 24 h. Thereafter, the cell proliferation reagent MTS (50 µL) was added to each well, and cells were incubated for 4 h. The absorbance was measured at 492 nm using a microplate reader (mean±SE, n = 3). **p<0.01 vs untreated group, #p<0.01 vs IS-treated group. HASMCs were transfected with or without PRR siRNA (20 nmol/L), and then serum starved for 24 h, followed by incubation with IS (250 µmol/L) or prorenin (20 nmol/L) for 24 h. Cell lysates were immunoblotted using anti-PRR and anti-tissue factor antibodies (**C–F**). Mean±SE (n = 3). *p<0.05, **p<0.01 vs untreated group, #p<0.05 vs IS-treated group. Ctrl: control.

IS promotes vascular smooth muscle cell proliferation through generation of ROS and transportation by OAT3 [33], [34]. Prorenin activates extracellular signal-regulated kinase (ERK) 1/2, leading to vascular smooth muscle cell proliferation, independent of Ang II generation [26], [27]. We examined whether IS-induced PRR is involved in proliferation of HASMCs. PRR siRNA suppressed IS-induced proliferation of HASMCs (Figure 7A). Prorenin (20 nmol/L) increased proliferation of HASMCs, whereas PRR siRNA suppressed prorenin-induced proliferation of HASMCs (Figure 7B). Thus, IS induces proliferation of HASMCs via prorenin/PRR pathway.

### IS-Induced PRR Activation is Involved in Tissue Factor Expression in Vascular Smooth Muscle Cells

IS is positively associated with tissue factor expression in CKD [35], [42]. The present study revealed that both IS and prorenin enhanced tissue factor protein expression in HASMCs. PRR siRNA suppressed IS-induced and prorenin-induced tissue factor expression in HASMCs (Figure 7D,E). Thus, IS induces tissue factor expression via prorenin/PRR pathway in HASMCs.

## Discussion

The novel findings of the present study are; 1) Aortic expression of PRR and prorenin/renin was increased in CKD rats, whereas AST-120 reduced their expression; 2) IS increased aortic expression of PRR and prorenin/renin in rats; 3) IS increased expression of PRR and prorenin through OAT3, ROS, AhR and NF-κB in vascular smooth muscle cells; 4) IS-induced PRR activation is involved in vascular smooth muscle cell proliferation; 5) IS-induced PRR activation is involved in tissue factor expression in vascular smooth muscle cells. Taken together, IS upregulates aortic expression of prorenin/PRR in vascular smooth muscle cells through OAT3-mediated uptake, ROS production, and activation of AhR and NF-κB p65. IS-induced activation of PRR is involved in cell proliferation and tissue factor expression in vascular smooth muscle cells.

We observed aortic expression of renin/prorenin in CKD rats and IS-administered rats. Further, IS induced prorenin expression in vascular smooth muscle cells. However, previous data demonstrated that vascular renin originates largely if not completely in the kidney [44], [45], and that the bulk of vascular renin is taken up from the circulation [46], [47]. Taken together, our observation might suggest that aortic expression of prorenin is induced in CKD rats and IS-induced rats.

ROS induced upregulation of PRR in diabetic rat kidneys [48]. IS induces ROS generation by increasing NADPH oxidase NOX-4 and through OAT3-mediated uptake in HASMCs [16], [17]. The present study revealed that NAC, DPI, and OAT3 siRNA suppressed IS-induced expression of PRR and prorenin in HASMCs. Thus, IS induced expression of PRR and prorenin through OAT3-mediated uptake and ROS production.

IS was identified as a potent endogenous ligand for AhR [40], [41]. IS induces activation and translocation of AhR in endothelial cells [41]. IS-induced activation of AhR upregulates NF-κB p65 expression in vascular smooth muscle cells (unpublished data). In the present study, both AhR siRNA and NF-κB p65 siRNA suppressed IS-induced expression of PRR and prorenin. Thus, IS-induced activation of AhR/NF-κB p65 pathway simulates the expression of PRR and prorenin in vascular smooth muscle cells.

Vascular smooth muscle cell proliferation is a key event in the pathogenesis of vascular complications. Binding of PRR with prorenin induced vascular smooth muscle cell proliferation via activation of ERK1/2, independent of angiotensin II [21], [22], [26], [27]. IS promoted aortic wall thickening and aortic calcification in hypertensive rats [14]. IS stimulated vascular smooth muscle cell proliferation through ROS production [22], and directly activated ERK1/2 [21]. In the present study, PRR siRNA suppressed both IS-induced and prorenin-induced cell proliferation in HASMCs. Thus, PRR is involved in IS-induced vascular smooth muscle cell proliferation.

Tissue factor is a mediator of injury-related thrombosis, and is elevated in the serum of advanced CKD patients. IS upregulates tissue factor expression in vascular smooth muscle cells [35] and endothelial cells [42]. The present study demonstrated that PRR siRNA suppressed IS-induced upregulation of TF expression in HASMCs. Thus, PRR is involved in IS-induced tissue factor expression in HASMCs. Taken together, IS-induced activation of prorenin-PRR pathway plays an important role in not only vascular smooth muscle cell proliferation but also tissue factor expression.

## References

[1] Foley RN, Parfrey PS, Sarnak MJ (1998) Clinical epidemiology of cardiovascular disease in chronic renal disease. Am J Kidney Dis 32: S112–119. 9820470 10.1053/ajkd.1998.v32.pm9820470

[2] Niwa T, Ise M (1994) Indoxyl sulfate, a circulating uremic toxin, stimulates the progression of glomerular sclerosis. J Lab Clin Med 124: 96–104. 8035108

[3] Niwa T (2010) Indoxyl sulfate is a nephro-vascular toxin. J Ren Nutr 20 Suppl 1: S2–S6. 20797565 10.1053/j.jrn.2010.05.002

[4] Niwa T (2011) Role of indoxyl sulfate in the progression of chronic kidney disease and cardiovascular disease: experimental and clinical effects of oral sorbent AST-120. Ther Apher Dial 15: 120–124. 21426500 10.1111/j.1744-9987.2010.00882.x

[5] Niwa T, Takeda N, Tatematsu A, Maeda K (1988) Accumulation of indoxyl sulfate, an inhibitor of drug-binding, in uremic serum as demonstrated by internal-surface reversed-phase liquid chromatography. Clin Chem 34: 2264–2267. 3141084

[6] Enomoto A, Takeda M, Tojo A, Sekine T, Cha SH, et al (2002) Role of organic anion transporters in the tubular transport of indoxyl sulfate and the induction of its nephrotoxicity. J Am Soc Nephrol 13: 1711–1720. 12089366 10.1097/01.asn.0000022017.96399.b2

[7] Shimizu H, Bolati D, Adijiang A, Muteliefu G, Enomoto A, et al (2011) NF-κB plays an important role in indoxyl sulfate-induced cellular senescence, fibrotic gene expression, and inhibition of proliferation in proximal tubular cells. Am J Physiol Cell Physiol 301: C1201–1212. 21832251 10.1152/ajpcell.00471.2010

[8] Shimizu H, Bolati D, Adijiang A, Enomoto A, Nishijima F, et al (2010) Senescence and dysfunction of proximal tubular cells are associated with activated p53 expression by indoxyl sulfate. Am J Physiol Cell Physiol 299: C1110–1117. 20720180 10.1152/ajpcell.00217.2010

[9] Shimizu H, Yisireyili M, Nishijima F, Niwa T (2012) Stat3 contributes to indoxyl sulfate-induced inflammatory and fibrotic gene expression and cellular senescence. Am J Nephrol 36: 184–189. 22889746 10.1159/000341515

[10] Miyazaki T, Ise M, Seo H, Niwa T (1997) Indoxyl sulfate increases the gene expression of TGF-β1, TIMP-1 and pro α(I) collagen in uremic rat kidneys. Kidney Int 52 Suppl 63: S15–22. 9350672

[11] Shimizu H, Bolati D, Higashiyama Y, Nishijima F, Shimizu K, et al (2012) Indoxyl sulfate upregulates renal expression of MCP-1 via production of ROS and activation of NF-κB, p53, ERK, and JNK in proximal tubular cells. Life Sci 90: 525–530. 22326498 10.1016/j.lfs.2012.01.013

[12] Shimizu H, Yisireyili M, Higashiyama Y, Nishijima F, Niwa T (2013) Indoxyl sulfate upregulates renal expression of ICAM-1 via production of ROS and activation of NF-κB and p53 in proximal tubular cells. Life Sci 92: 143–148. 23201429 10.1016/j.lfs.2012.11.012

[13] Barreto FC, Barreto DV, Liabeuf S, Meert N, Glorieux G, et al (2009) Serum indoxyl sulfate is associated with vascular disease and mortality in chronic kidney disease patients. Clin J Am Soc Nephrol 4: 1551–1558. 19696217 10.2215/CJN.03980609 PMC2758258

[14] Adijiang A, Goto S, Uramoto S, Nishijima F, Niwa T (2008) Indoxyl sulphate promotes aortic calcification with expression of osteoblast-specific proteins in hypertensive rats. Nephrol Dial Transplant 23: 1892–1901. 18334529 10.1093/ndt/gfm861

[15] Tumur Z, Shimizu H, Enomoto A, Miyazaki H, Niwa T (2010) Indoxyl sulfate upregulates expression of ICAM-1 and MCP-1 by oxidative stress-induced NF-kB activation. Am J Nephrol 31: 435–441. 20389059 10.1159/000299798

[16] Adelibieke Y, Shimizu H, Saito S, Mironova R, Niwa T (2013) Indoxyl sulfate counteracts endothelial effects of erythropoietin through suppression of Akt phosphorylation. Circ J 77: 1326–1336. 23337206 10.1253/circj.cj-12-0884

[17] Muteliefu G, Enomoto A, Jiang P, Takahashi M, Niwa T (2009) Indoxyl sulphate induces oxidative stress and the expression of osteoblast-specific proteins in vascular smooth muscle cells. Nephrol Dial Transplant 24: 2051–2058. 19164326 10.1093/ndt/gfn757

[18] Muteliefu G, Shimizu H, Enomoto A, Nishijima F, Takahashi M, et al (2012) Indoxyl sulfate promotes vascular smooth muscle cell senescence with upregulation of p53, p21 and prelamin A through oxidative stress. Am J Physiol Cell Physiol 303: C126–134. 22555846 10.1152/ajpcell.00329.2011

[19] Lekawanvijit S, Kompa AR, Wang BH, Kelly DJ, Krum H (2012) Cardio-renal syndrome: the emerging role of protein-bound uremic toxins. Circ Res 111: 1470–1483. 23139286 10.1161/CIRCRESAHA.112.278457

[20] Yisireyili M, Shimizu H, Saito S, Enomoto A, Nishijima F, et al (2013) Indoxyl sulfate promotes cardiac fibrosis with enhanced oxidative stress in hypertensive rats. Life Sci 92: 1180–1185. 23702423 10.1016/j.lfs.2013.05.008

[21] Nguyen G, Muller DN (2010) The biology of the (pro)renin receptor. J Am Soc Nephrol 21: 18–23. 19917780 10.1681/ASN.2009030300

[22] Greco CM, Camera M, Facchinetti L, Brambilla M, Pellegrino S, et al (2012) Chemotactic effect of prorenin on human aortic smooth muscle cells: a novel function of the (pro)renin receptor. Cardiovasc Res 95: 366–374. 22721990 10.1093/cvr/cvs204

[23] Batenburg WW, Lu X, Leijten F, Maschke U, Müller DN, et al (2011) Renin- and prorenin-induced effects in rat vascular smooth muscle cells overexpressing the human (pro)renin receptor: does (pro)renin-(pro)renin receptor interaction actually occur? Hypertension 58: 1111–1119. 22025376 10.1161/HYPERTENSIONAHA.111.180737

[24] Huang Y, Wongamorntham S, Kasting J, McQuillan D, Owens RT, et al (2006) Renin increases mesangial cell transforming growth factor-beta1 and matrix proteins through receptor-mediated, angiotensin II-independent mechanisms. Kidney Int 69: 105–113. 16374430 10.1038/sj.ki.5000011

[25] Saito S, Shimizu H, Yisireyili M, Nishijima F, Enomoto A, et al (2014) Indoxyl sulfate-induced activation of (pro)renin receptor is involved in expression of transforming growth factor-β1 and α-smooth muscle actin in proximal tubular cells. Endocrinology en20131937. 10.1210/en.2013-1937 24601883

[26] Sakoda M, Ichihara A, Kaneshiro Y, Takemitsu T, Nakazato Y, et al (2007) (Pro)renin receptor-mediated activation of mitogen-activated protein kinases in human vascular smooth muscle cells. Hypertens Res 30: 1139–1146. 18250563 10.1291/hypres.30.1139

[27] Liu G, Hitomi H, Hosomi N, Shibayama Y, Nakano D, et al (2011) Prorenin induces vascular smooth muscle cell proliferation and hypertrophy via epidermal growth factor receptor-mediated extracellular signal-regulated kinase and Akt activation pathway. J Hypertens 29: 696–705. 21252698 10.1097/HJH.0b013e328343c62b

[28] Cruciat CM, Ohkawara B, Acebron SP, Karaulanov E, Reinhard C, et al (2010) Requirement of prorenin receptor and vacuolar H^+^-ATPase-mediated acidification for Wnt signaling. Science 327: 459–463. 20093472 10.1126/science.1179802

[29] Oshima Y, Kinouchi K, Ichihara A, Sakoda M, Kurauchi-Mito A, et al (2011) Prorenin receptor is essential for normal podocyte structure and function. J Am Soc Nephrol 22: 2203–2212. 22052048 10.1681/ASN.2011020202 PMC3279932

[30] Nguyen G, Muller DN (2010) The biology of the (pro)renin receptor. J Am Soc Nephrol 21: 18–23. 19917780 10.1681/ASN.2009030300

[31] Kinouchi K, Ichihara A, Sano M, Sun-Wada GH, Wada Y, et al (2010) The (pro)renin receptor/ATP6AP2 is essential for vacuolar H^+^-ATPase assembly in murine cardiomyocytes. Circ Res 107: 30–34. 20570919 10.1161/CIRCRESAHA.110.224667

[32] Riediger F, Quack I, Qadri F, Hartleben B, Park JK, et al (2011) Prorenin receptor is essential for podocyte autophagy and survival. J Am Soc Nephrol 22: 193–202. 10.1681/ASN.2011020200 PMC3279931 22034640

[33] Yamamoto H, Tsuruoka S, Ioka T, Ando H, Ito C, et al (2006) Indoxyl sulfate stimulates proliferation of rat vascular smooth muscle cells. Kidney Int 69: 1780–1785. 16612331 10.1038/sj.ki.5000340

[34] Muteliefu G, Enomoto A, Niwa T (2009) Indoxyl sulfate promotes proliferation of human aortic smooth muscle cells by inducing oxidative stress. J Ren Nutr 19: 29–32. 19121767 10.1053/j.jrn.2008.10.014

[35] Chitalia VC, Shivanna S, Martorell J, Balcells M, Bosch I, et al (2013) Uremic serum and solutes increase post-vascular interventional thrombotic risk through altered stability of smooth muscle cell tissue factor. Circulation 127: 365–376. 23269489 10.1161/CIRCULATIONAHA.112.118174 PMC4407990

[36] Takii Y, Figueiredo AF, Inagami T (1985) Application of immunochemical methods to the identification and characterization of rat kidney inactive renin. Hypertension 7: 236–243. 3884504 10.1161/01.hyp.7.2.236

[37] Bolati D, Shimizu H, Niwa T (2012) AST-120 ameliorates epithelial-to-mesenchymal transition and interstitial fibrosis in the kidneys of chronic kidney disease rats. J Ren Nutr 22: 176–180. 22200438 10.1053/j.jrn.2011.10.015

[38] Adijiang A, Shimizu H, Higuchi Y, Nishijima F, Niwa T (2011) Indoxyl sulfate reduces klotho expression and promotes senescence in the kidneys of hypertensive rats. J Ren Nutr 21: 105–109. 21195930 10.1053/j.jrn.2010.10.020

[39] Niwa T, Nomura T, Sugiyama S, Miyazaki T, Tsukushi S, et al (1997) The protein metabolite hypothesis, a model for the progression of renal failure: An oral adsorbent lowers indoxyl sulfate levels in undialyzed uremic patients. Kidney Int 52 Suppl 62: S23–28. 9350673

[40] Schroeder JC, Dinatale BC, Murray IA, Flaveny CA, Liu Q, et al (2010) The uremic toxin 3-indoxyl sulfate is a potent endogenous agonist for the human aryl hydrocarbon receptor. Biochemistry 49: 393–400. 20000589 10.1021/bi901786x PMC2805781

[41] Watanabe I, Tatebe J, Namba S, Koizumi M, Yamazaki J, et al (2013) Activation of aryl hydrocarbon receptor mediates indoxyl sulfate-induced monocyte chemoattractant protein-1 expression in human umbilical vein endothelial cells. Circ J 77: 224–230. 23037589 10.1253/circj.cj-12-0647

[42] Gondouin B, Cerini C, Dou L, Sallée M, Duval-Sabatier A, et al (2013) Indolic uremic solutes increase tissue factor production in endothelial cells by the aryl hydrocarbon receptor pathway. Kidney Int 84: 733–744. 23636172 10.1038/ki.2013.133

[43] Sallée M, Dou L, Cerini C, Poitevin S, Brunet P, et al (2014) The aryl hydrocarbon receptor-activating effect of uremic toxins from tryptophan metabolism: a new concept to understand cardiovascular complications of chronic kidney disease. Toxins 6: 934–949. 24599232 10.3390/toxins6030934 PMC3968369

[44] Inagami T, Murakami T, Higuchi K, Nakajo S (1991) Role of vascular wall renin: intracellular and extracellular mechanism. Blood Vessels 28: 217–223. 2001472 10.1159/000158865

[45] Kato H, Iwai N, Inui H, Kimoto K, Uchiyama Y, et al (1993) Regulation of vascular angiotensin release. Hypertension 21: 446–454. 8458646 10.1161/01.hyp.21.4.446

[46] Hilgers KF, Hilgenfeldt U, Veelken R, Muley T, Ganten D, et al (1993) Angiotensinogen is cleaved to angiotensin in isolated rat blood vessels. Hypertension 21: 1030–1034. 8505088 10.1161/01.hyp.21.6.1030

[47] Müller DN, Luft FC (1998) The renin-angiotensin system in the vessel wall. Basic Res Cardiol 93 Suppl 2: 7–14. 9833156 10.1007/s003950050194

[48] Siragy HM, Huang J (2008) Renal (pro)renin receptor upregulation in diabetic rats through enhanced angiotensin AT1 receptor and NADPH oxidase activity. Exp Physiol 93: 709–714. 18192338 10.1113/expphysiol.2007.040550 PMC2586037

